# Applications of the Mittag-Leffler law to linear kinetic models & diffusion equations

**DOI:** 10.1038/s41598-025-00967-7

**Published:** 2025-05-13

**Authors:** Victor Tebogo Monyayi, Emile Franc Doungmo Goufo, Ignace Tchangou Toudjeu

**Affiliations:** 1 Department of Mathematical Sciences, University of South Africa, Florida, 0003 South Africa; 2 Department of Electrical Engineering, Tshwane University of Technology, Pretoria, 0183 South Africa

**Keywords:** Atangana–Baleanu–Caputo fractional derivative, Diffusion equations, Sumudu transform, Homotopy perturbation method, Mathematics and computing, Physics

## Abstract

In this paper, we find the solutions to kinetic models and a one-dimensional diffusion equation applied to the Atangana-Baleanu-Caputo fractional derivative (ABCFD). The homotopy perturbation method is combined with the Sumudu transform of the Atangana-Baleanu fractional derivative in the Caputo sense to form a modified technique to solve two and three-dimensional diffusion equations. The technique yields a solution in the form of a power series that contains easily computable terms that converge to the exact solution. We observe that when we increase the number of computing terms, the series becomes closer to the exact solution so that the absolute error between the exact solution and the approximate solution becomes very small. Mathematica software is utilized to perform the graphical representations of the exact and approximate solutions. These solutions are analyzed by manipulating the variables to observe their effect on each other. One of our objectives is to show that if we let the fractional order be one, we always obtain the solution of the traditional derivative. The approximate solutions of the ABCFD models are compared with the approximate solutions of the classical Caputo fractional derivative (CFD). Our results indicate that the classical Caputo method converges to the exact solution more rapidly than the Atangana–Baleanu–Caputo method, suggesting that the classical Caputo method may be more suitable for applications requiring rapid convergence, while the ABC method may be suitable for non-local behaviors and other specific contexts where its unique properties offer advantages.

## Introduction

Fractional derivatives and integrals are the generalizations of classical calculus. They allow us to describe non-local and memory-dependent behavior in systems. The classical integer-order calculus cannot capture such complexities. There exist many kinds of fractional derivatives in the literature, but the most used are the Caputo, Riemann–Liouville, and Caputo–Fabrizio fractional derivatives. However, the Caputo and Riemann-Liouville fractional derivatives seem to be particularly suitable for describing physical phenomena, associated with damage, fatigue and electromagnetic hysteresis but are not capable of properly describing some behavior observed in materials with huge heterogeneities and structures with different scales^1^. The Caputo-Fabrizio fractional derivative has a non-singular kernel but it does not have a non-local kernel, its kernel depends only on the difference $(t - \tau )$, which makes it local. In 2016, Atangana and Baleanu proposed a novel approach to fractional derivatives based on the generalized Mittag-Leffler function. Their goal was to introduce fractional differential operators with non-singular and non-local kernels. These operators have inspired significant research in fractional calculus because they can provide excellent descriptions due to their Mittag-Leffler memory. However, critics argue that fractional derivatives with non-singular kernels, such as the ABC fractional derivative, do not satisfy certain fundamental properties of fractional calculus. For example, they may not admit the existence of a corresponding convolution integral, which is a key aspect of traditional fractional derivatives like the Caputo or Riemann-Liouville derivatives^2^. Furthermore, for these non-singular kernel derivatives, the derivative’s value at the start time $t=0$ is always zero, which may impose unnatural restrictions on the differential equations and models that employ them.^2^.Despite these critics, The ABC fractional derivative has been shown to be effective in modeling complex systems due to its ability to capture memory and hereditary properties more accurately than the classical Caputo fractional derivative. Complex models using the ABC fractional derivative can be found in various studies, such as those addressing boundary value problems of hybrid fractional differential equations^3^, non-linear fractional models^4^ and intricate dynamics of alcohol consumption^5^. In this paper, we discuss the fractional linear kinetic models and fractional diffusion equations. The type of fractional differential operator we will use is the Caputo fractional derivative with a Mittag-Leffler kernel, also known as the Atangana-Baleanu-Caputo fractional derivative (ABCFD). We find the solutions to the models and compare them with the solutions of the classical Caputo fractional derivative.

Linear fractional differential kinetic models are commonly used to describe reaction rates in various chemical processes. They can also be used to describe radioactive decay, and model population growth and decay. Diffusion equations are utilized to model the movement of magnetic fields and plasma in astrophysical environments to assist in understanding phenomena like star formation and the behavior of interstellar matter^6^. They are also applied in modeling chemical reactions, and heat transfer and are essential for explaining how medications, nutrients and other substances are transported through biological tissues^7,8^. In addition to heat transfer, a novel approximation method for two-dimensional phase transfer problems in a moving domain with a heat generation parameter has been explored by Joshi et al^9^.

A diffusion equation is an equation that is in partial differential form, that describes the distribution of a quantity (such as heat, particles, or chemicals) over space and time. A mathematical equation that contains two or more independent variables is known as a partial differential equation (PDE). Many physical problems are modeled using differential equations with their given initial conditions. Since it is difficult to find the exact solutions of some partial differential equations (PDEs), researchers have established some methods of finding the best approximate solutions by numerical methods and series solution methods. Such methods are also important in finding the exact and approximate solutions of fractional linear and nonlinear differential equations such as diffusion equations. Adomian decomposition method (ADM), iterative method (IM) and homotopy perturbation method (HPM) are the famous and powerful methods to solve the above-mentioned equations, their efficiency is well known with to process rapid convergency of the solution and only after few iterations they reach their desired accuracy. These methods can be coupled with the Laplace or Sumudu transforms, for instance, if the iterative method (IM) is coupled with the Laplace transform, it will be called the iterative Laplace transform method (ILTM) and if combined with the Sumudu transform, it will be called the iterative Sumudu transform method (ISTM). These methods have been applied in^10–13^ to find the exact and approximate solutions of the fractional Navier-Stokes system that describes the motion of a viscous fluid in a tube and they produced the same solutions when the fractional order $\varpi =1$, even if different fractional differential operators are used. In^14^ the authors solved the fractional Navier-Stokes system by coupling the Laplace transform with the residual power series method and also got the same results when $\varpi = 1$. Other methods such as the Laplace decomposition technique, the reproducing kernel Hilbert space technique, the variational iteration technique, the local fractional variational iteration technique, the fractional complex transform technique and the generalized differential transform technique are all mentioned in^15^. In^16^ the authors used a hybrid approach to solve and calculate the approximate solutions of a two-dimensional time-fractional Cattaneo model with Riesz distributed-order space-fractional operator.

Motivated by the above works of others, this paper is structured as follows: In Sect. 2, we provide the definitions and theorems regarding the Caputo fractional derivative (CFD) and Atangana-Baleanu-Caputo fractional derivative (ABCFD), which we are going to apply in the next sections. In Sect. 3, we find the solutions of the three fractional kinetic models and comment on the solutions. In Sect. 4, we compare the solutions of the classical Caputo fractional derivative (CFD) stationary models with the solutions of the ABCFD stationary models. In Sect. 5, we solve a one-dimensional fractional diffusion equation using the separation of variables method. In Sect. 6, we solve fractional diffusion equations by coupling the Sumudu transform with the homotopy perturbation method (HPM) to form the modified homotopy perturbation Sumudu transform method (HPSTM). The advantage of this method is that we combine the two methods, the Sumudu transform and the homotopy perturbation method (HPM) which are both very important in finding the solutions of linear and nonlinear fractional differential equations. Furthermore, the type of Sumudu transform is of the Caputo fractional derivative with a Mittag-Leffler kernel, which exhibits good memory effects when applied to the modeling of complex systems, such as viscoelastic materials, anomalous diffusion processes, and various engineering problems. We also compare this model with the Caputo fractional derivative model.

### Main results

We study the diffusion model to explore the comparative performance of the ABC fractional derivative and the classical Caputo fractional derivative in approximating solutions to fractional differential equations. The motivation is to understand the strengths and limitations of each method in capturing the dynamics of systems with memory and hereditary properties, which are prevalent in various scientific and engineering applications. We consider the model of a two-dimensional fractional diffusion equation of the form

1 $$\begin{aligned} \frac{{\partial }^{\varpi } {u(x,y,t)} }{\partial t^{\varpi }}=c\left( \frac{{\partial }^{2} {u(x,y,t)} }{\partial x^2}+\frac{{\partial }^{2} {u(x,y,t)} }{\partial y^2}\right) ,\quad 0< \varpi \le 1,\quad 0\le x,y\le 1,\quad t>0, \end{aligned}$$

which is subject to the following initial condition

2 $$\begin{aligned} u(x,y, 0)=f(x,y), \end{aligned}$$

where $\varpi$ is the Atangana-Baleanu-Caputo fractional order, *u*(*x*, *y*, *t*) is the concentration of the diffusing substance (or the quantity of interest such as temperature or concentration), *c* is the diffusion coefficient, *x* and *y* are the spatial coordinates, and *t* is time. We will assume that the diffusion coefficient *c* is equal to one when doing our analysis.

Then, we intend to find the exact and approximate solutions of the diffusion equation (1) with its initial condition (2) using the modified HPSTM. We apply the Sumudu transform of the ABCFD on both sides of Eq. (1) and use the homotopy perturbation method to expand the solution into a power series. We use Mathematica software to draw the 2D and 3D graphs of the solutions that we obtained. We set the value of a fractional order $\varpi$ to be one, and other variables are manipulated so that we can be able to see their relationships. One of our objectives will be to show that all fractional derivatives are the generalization of the classical (normal) derivative, for instance, if we let the fractional order be 1, then, all solutions will lead us to the classical solutions, but they will give us different results if we choose other fractional values between 0 and 1. We also plot the 2Ds graphs of 3rd term approximation solutions for different values of fractional parameter $\varpi$ (0.85, 0.90, 0.95) and observe how close they are to the exact solution. The absolute errors between the exact and the approximate solutions are calculated and compared at the 10*th* approximations. We also do the same with the three-dimensional fractional diffusion equation and compare the results with the results of the Caputo fractional derivative models.

## Preliminaries

In this section, we provide important theorems and definitions of the classical Caputo and Atangana-Baleanu-Caputo fractional calculus.

### Definition 1

^11,24,31^ Assume $u\in {H}^{1}(a, b), \varpi \in {[0, 1], a<b }$; therefore, the Caputo fractional derivative with the power law kernel, is provided by

3 $$\begin{aligned} ^{CFD}_{a}D^{\varpi }_tu(t)=\frac{1}{\Gamma (1-\varpi )}\int _a^t\dot{u}(\tau ) (t-\tau )^{-\varpi }d \tau . \end{aligned}$$

### Theorem 1

^11,24,31^ *The fractional integral of the classical Caputo fractional derivative is given as*

4 $$\begin{aligned} {^{CFI}} I^{\varpi }_tu(t) =\frac{1}{\Gamma (\varpi )}\int _0^t(t-{\tau })^{\varpi -1}u(\tau )d{\tau }. \end{aligned}$$

### Theorem 2

^10,11^ *The Laplace transform of the Caputo fractional derivative with the power law kernel is given as*

5 $$\begin{aligned} \mathcal {L} [{^{CFD}_{0}D^{\varpi }_tu(t)}](s)= s^{\varpi }\mathcal {L}[u(t)](s)-s^{\varpi -1}u(0). \end{aligned}$$

### Theorem 3

^17,31^ *The Sumudu transform of the classical Caputo fractional derivative is specified as*

6 $$\begin{aligned} S\left( ^{CFD}D^{\varpi }_t u(t)\right) =\frac{1}{ s^{\varpi }} (S(u)-u(0)). \end{aligned}$$

### Definition 2

^18–23^ Assume $u\in {H}^{1}(a, b), \varpi \in {[0, 1], a<b }$; therefore, the Caputo fractional derivative with a Mittag-Leffler kernel, is provided by

7 $$\begin{aligned} ^{ABCFD}_{a}D^{\varpi }_tu(t)=\frac{B({\varpi })}{(1-\varpi )}\int _a^t\dot{u}(\tau ) E_{\varpi }\left( -\frac{\varpi (t-\tau )}{1-\varpi }^{\varpi }\right) d \tau , \end{aligned}$$

where $B(\varpi )=1-\varpi +\frac{\varpi }{\Gamma {(\varpi )}}$ so that $B(1)=B(0)=1$ and $E_{\varpi }$ is the Mittag-Leffler function given as follows:

### Definition 3

^21,24–26^ For every $X \in \mathbb {C}$, the Mittag-Leffler function (MLF) with parameter $\varpi$ is provided by

8 $$\begin{aligned} E_{\varpi }(X)=\sum ^{\infty }_{n=0}\frac{X^{n}}{\Gamma (\varpi n+1)},\quad \quad {\varpi }>0, \end{aligned}$$

and with two parameters is defined as

9 $$\begin{aligned} E_{\varpi ,\vartheta }(X)=\sum ^{\infty }_{n=0}\frac{X^{n}}{\Gamma (\varpi n+\vartheta )}, \quad \quad {\varpi }>0, {\vartheta } >0 \quad X \in \mathbb {C}. \end{aligned}$$

The Mittag-Leffler function (MLF) is the generalization of the exponential function. If $\varpi =1$ in Eq. (8) and $\varpi =\vartheta =1$ in Eq. (9), we obtain $E_{1}(X)=E_{1, 1}=e^{X}$, meaning that we recover the classical exponential function $e^{X}$.

### Theorem 4

^18,20,22^ *The Laplace transform of the Caputo fractional derivative with a Mittag-Leffler kernel is given as*

10 $$\begin{aligned} \mathcal {L} [{^{ABCFD}_{0}D^{\varpi }_tu(t)}](s)=\frac{B({\varpi })}{1-\varpi } \frac{ s^{\varpi }\mathcal {L}[u(t)](s)-s^{\varpi -1}u(0)}{s^\varpi +\frac{\varpi }{1-\varpi }}. \end{aligned}$$

### Theorem 5

^20^ *The Sumudu transform of the Caputo fractional derivative with a Mittag-Leffler kernel is specified as follows*

11 $$\begin{aligned} S\left( ^{ABCFD}D^{\varpi }_t u(t)\right) =\frac{B(\varpi )}{1-\varpi (1- s^{\varpi })} (S(u)-u(0)). \end{aligned}$$

### Theorem 6

^18,23^ *The fractional integral of the Atangana-Baleanu fractional derivative is given as follows*

12 $$\begin{aligned} {^{ABFI}} I^{\varpi }_tu(t) =\frac{1-\varpi }{B(\varpi )}u(t)+\frac{\varpi }{B(\varpi )\Gamma (\varpi )}\int _0^t(t-{\tau })^{\varpi -1}u(\tau )d{\tau }. \end{aligned}$$

### Lemma 1

*Let* *u* *be the solution of the following fractional differential equation*

$${^{ABCFD}} D^{\varpi }_tu(t) = 0,$$

*for* $0<\varpi <1$ and $t \ge 0,$ *then* *u* *is a constant function*.

## Application of ABCFD to kinetic models

In this section, we discuss the three linear fractional kinetic models and provide some explanations and their applications to real life.

### First model (stationary model)

Consider the first stationary model in the form of the ABCFD

13 $$\begin{aligned} {\left\{ \begin{array}{ll} \begin{aligned} & ^{ABCFD} D^{\varpi }_tu(t) = 0, \;\;\;0<\varpi \le 1,\;\;t>0, \\ & u(0)= \,u_o. \end{aligned} \end{array}\right. } \end{aligned}$$

If we apply the Laplace transform (10) on both sides of the first model (13) we obtain

$$\frac{B({\varpi })}{1-\varpi } \frac{ s^{\varpi }\mathcal {L}[u(t)](s)-s^{\varpi -1}u(0)}{s^\varpi +\frac{\varpi }{1-\varpi }}=0.$$

**Fig. 1:**
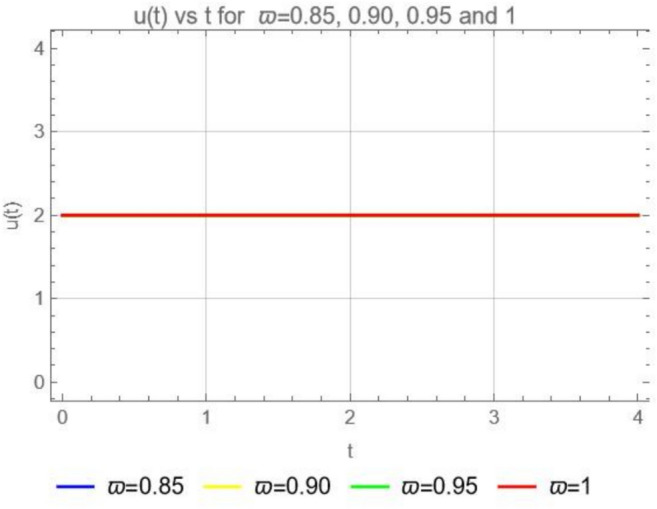
Numerical solution of (13) with the initial condition $u_0=2$ for $\varpi = 0.85,$ 0.90,  0.95 and 1.

So that $\bar{u}(s)=\frac{u_0}{s}$ and if we apply the inverse Laplace transform $\mathcal {L}^{-1} [\bar{u}(s),t]=u_o$ $\forall t>0$ and yields

$$u(t)=u_{0}=C,$$

where C is a constant. Moreover, the solution $u(t)=u_{0}=C,$ is the direct proof of lemma (1). Figure 1, is the graphical representation for the solutions of (13) and it illustrates the constant results.

We can see in Fig. 1 that the solution to (13) represents a system in equilibrium where no change occurs over time.

### Second model

Consider the second model in the form of the ABCFD

14 $$\begin{aligned} {\left\{ \begin{array}{ll} \begin{aligned} & ^{ABCFD} D^{\varpi }_tu(t) = K, \;\;\;0<\varpi \le 1,\;\;t>0, \\ & u(0)= \,u_o. \end{aligned} \end{array}\right. } \end{aligned}$$

If we apply the Laplace transform (10) on both sides of the second model (14) we obtain

$$\frac{B({\varpi })}{1-\varpi } \frac{ s^{\varpi }\mathcal {L}[u(t)](s)-s^{\varpi -1}u(0)}{s^\varpi +\frac{\varpi }{1-\varpi }}=K\mathcal {L} [1, s],$$

and solving for $\mathcal {L}[u(t)](s)$ yields

$$\mathcal {L}[u(t)](s)=\frac{u_0}{s}+\frac{K(1-\varpi )}{B(\varpi )}\mathcal {L} [1, s]+\frac{K\varpi }{B(\varpi )}\frac{\mathcal {L} [1, s]}{s^{\varpi }}.$$

Then applying the inverse Laplace transform we get

15 $$\begin{aligned} u(t)=u_0+\frac{K(1-\varpi )}{B(\varpi )}+\frac{K}{B(\varpi )}\frac{t^{\varpi }}{\Gamma {(\varpi )}}. \end{aligned}$$

The solution (15) is equivalent to

$$u(t)=u_o+\frac{1-\varpi }{B(\varpi )}K+\frac{\varpi {K}}{B(\varpi )\Gamma (\varpi )}\int _0^t(t-{\tau })^{\varpi -1}d{\tau },$$

when we apply the anti-derivative on both sides of (14). Furthermore in order to obtain an average equal to 1, we consider

$$\frac{1-\varpi }{B(\varpi )}+\frac{\varpi }{{B(\varpi )}\Gamma {(\varpi )}}=1,$$

which leads to

$${B(\varpi )}=1-\varpi +\frac{\varpi }{\Gamma {(\varpi )}}.$$

**Fig. 2:**
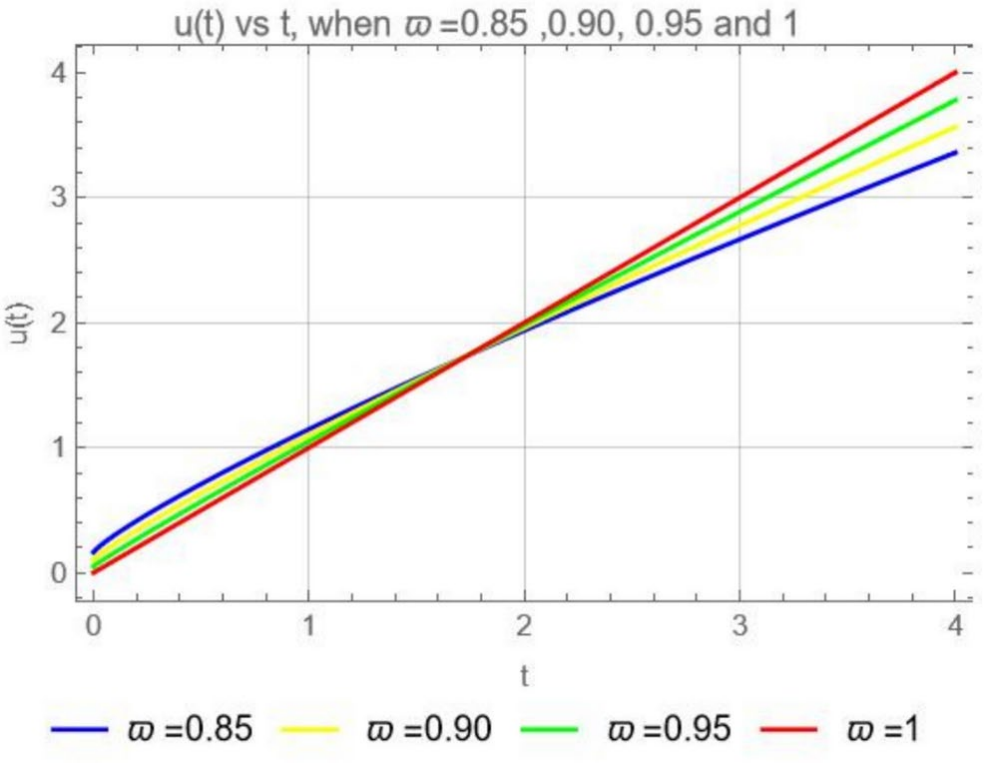
Numerical solution of (14) with the initial condition $u_0=0$, when $K=1$, for $\varpi = 0.85$, 0.90, 0.95 and 1.

(i) . When $K=0,$ we recover from (15) the solution $u(t)=u_0=C$.
(ii) . When $\varpi =1,$ we get from (15) the well-known classical solution: $u(t)=u_{o} +Kt$. A graphical representation for the solution (15) of the second model (14) is given in Fig. 2.

The solution (15) represents a system where *u* increases linearly with time *t* (this indicates a linear relationship between *u* and *t*).

### Fractional relaxation model

Consider the third model in the form of the ABCFD

16 $$\begin{aligned} {\left\{ \begin{array}{ll} \begin{aligned} & ^{ABCFD} D^{\varpi }_tu(t) = -Ku, \;\;\;0<\varpi \le 1,\;\;t>0, \\ & u(0)= \,u_o. \end{aligned} \end{array}\right. } \end{aligned}$$

If we apply the Laplace transform (10) on both sides of the third model (16) we obtain

$$\frac{B({\varpi })}{1-\varpi } \frac{ s^{\varpi }\mathcal {L}[u(t)](s)-s^{\varpi -1}u(0)}{s^\varpi +\frac{\varpi }{1-\varpi }}=-K\mathcal {L}[u(t)](s),$$

and solving for $\mathcal {L}[u(t)](s)$ yields

$$\mathcal {L}[u(t)](s)= \frac{u_o B(\varpi )s^{\varpi -1}}{s^{\varpi }(B(\varpi )+K-K\varpi )+K\varpi }.$$

Applying the inverse Laplace transform we get

17 $$\begin{aligned} u(t)=\frac{u_o B(\varpi )}{B(\varpi )+K-K\varpi }\mathcal {L}^{-1}\left[ \frac{s^{\varpi -1}}{s^{\varpi }+\frac{K\varpi }{B(\varpi )+K-K\varpi }}\right] . \end{aligned}$$

Since the Laplace transform of the Mittag-Leffler function is given by

18 $$\begin{aligned} \mathcal {L}\left[ t^{\alpha -1}E_{{\varpi }, {\alpha }}( \lambda t^{\varpi })\right] (s)=\frac{s^{\varpi -\alpha }}{s^\varpi -\lambda }, \end{aligned}$$

if $\alpha =1$ we have

19 $$\begin{aligned} \mathcal {L}[E_{{\varpi }, {1}}( \lambda t^{\varpi })](s)=\frac{s^{\varpi -1}}{s^\varpi -\lambda }, \end{aligned}$$

then it is clear that in (17)

$$\lambda =\frac{K\varpi }{B(\varpi )+K-K\varpi },$$

so that solution in (17) when using (19) it becomes,

20 $$\begin{aligned} u(t)=\frac{u_o B(\varpi )}{B(\varpi )+K-K\varpi } E_{{\varpi }, {1}}\left( \frac{-K\varpi t^{\varpi }}{B(\varpi )+K-K\varpi }\right) . \end{aligned}$$

(i) . When $\varpi =1,$ we obtain from Eq. (20) the classical solution $u(t)=u_0 E_{1,1} (-Kt)=u_0e^{-Kt}.$
(ii) . When $K=0,$ we obtain (from Eq. (20))
  $$u(t)=u_0E_{\varpi }(0)=u_0\sum ^{\infty }_{n=0}\frac{0^{n}}{\Gamma (\varpi n+1)},$$

**Fig. 3:**
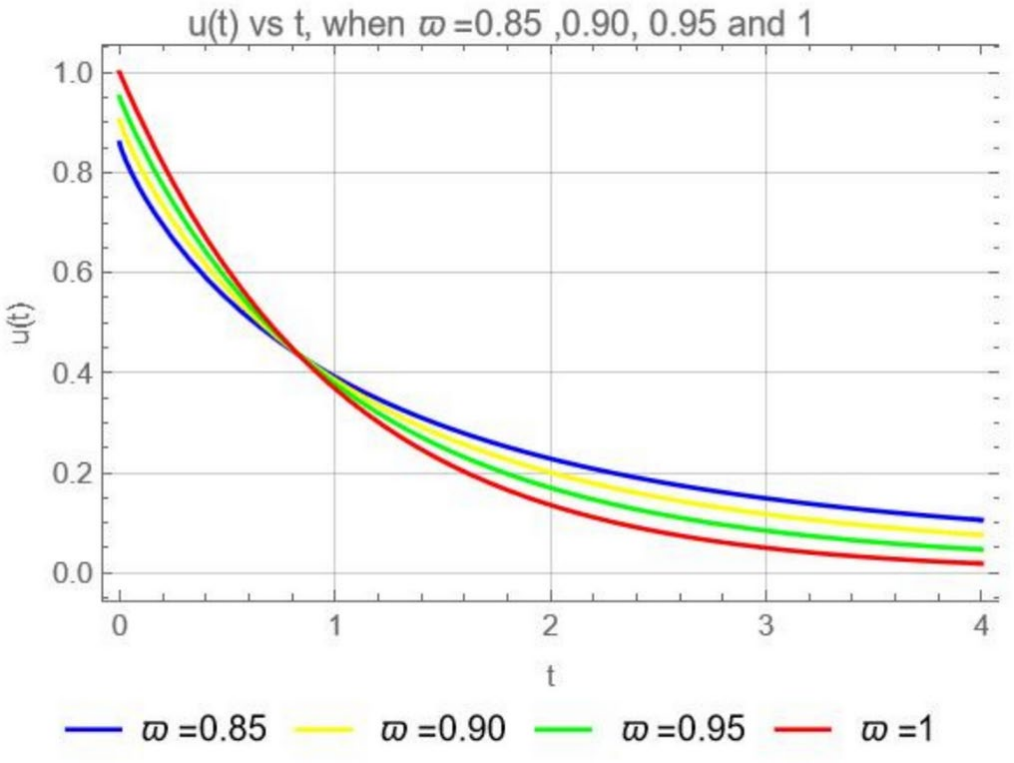
Numerical solution (20) with the initial condition $u_0=1$ when $K=1$, $\varpi =0.85,$ 0.90, 0.95 and 1.

For $n=0,$ the first term of *u*(*t*) is $u_0\frac{0^0}{\Gamma (1)}=u_0\frac{0^0}{0!}=u_0.1.$ For $n>0$, all terms are zero, since $0^n=0$ for all $n>0.$ So that when $K=0$, we have $u(t)=u_0$. The solution (20) represents a system where *u* decreases over time (when $K>0$), such as cooling processes or radioactive decay with the impact of the memory effect of the Mittag-Leffler kernel so that the current state depends on the entire history. A graphical representation for the solution (20) of the third model (16) is given in Fig. 3.

In addition, from Eq. (18), we have the following Lemma.

#### Lemma 2

*Let* $\varpi$, $\alpha >0$, $\lambda \in \mathbb {R}$, *and* $s^{\varpi }>|\lambda |$, *then we have the following inverse Laplace transform*

21 $$\begin{aligned} \mathcal {L}^{-1}\left[ \frac{s^{\varpi -\alpha }}{s^\varpi +\lambda }\right] =t^{\alpha -1}E_{{\varpi }, {\alpha }}(- \lambda t^{\varpi }). \end{aligned}$$

#### Proof

By using the series expansion we can rewrite $\frac{s^{\varpi -\alpha }}{s^\varpi +\lambda }$ as

$$\frac{s^{\varpi -\alpha }}{s^\varpi +\lambda }=\frac{1}{s^{\alpha }}\frac{1}{1+\frac{\lambda }{s^{\varpi }}}= \frac{1}{s^{\alpha }}\sum _{n=0}^{\infty }\left( \frac{-{\lambda }}{s^{\varpi }}\right) ^n=\sum _{n=0}^{\infty }\frac{(-\lambda )^n}{s^{n\varpi +\alpha }}.$$

Then, the inverse Laplace transform of the above function is

$$\sum _{n=0}^{\infty }\frac{(-\lambda )^n t^{n\varpi +\alpha -1}}{\Gamma {(n\varpi +\alpha )}}= t^{\alpha -1}\sum _{n=0}^{\infty }\frac{(-\lambda t^{\varpi })^n }{\Gamma {(n\varpi +\alpha )}}=t^{\alpha -1}E_{{\varpi }, {\alpha }}(- \lambda t^{\varpi }).$$

$$\square$$

## Comparison of the classical Caputo fractional derivative (CFD) and the ABCFD stationary models

Consider the first stationary model in the form of the CFD

22 $$\begin{aligned} {\left\{ \begin{array}{ll} \begin{aligned} & ^{CFD} D^{\varpi }_tu(t) = 0, \;\;\;0<\varpi \le 1,\;\;t>0, \\ & u(0)= \,u_o. \end{aligned} \end{array}\right. } \end{aligned}$$

If we apply the Laplace transform (5) on both sides of the first model (22) we obtain

$$s^{\varpi }\mathcal {L}[u(t)](s)-s^{\varpi -1}u(0)=0.$$

So that $\bar{u}(s)=\frac{u_0}{s}$ and if we apply the inverse Laplace transform $\mathcal {L}^{-1} [\bar{u}(s),t]=u_o$ $\forall t>0$ and yields

$$u(t)=u_{0}=C,$$

where C is a constant. The graphical representation for the solutions of (22) is the same as the one for the solutions of (13).

### Second model

Consider the second model in the form of the CFD

23 $$\begin{aligned} {\left\{ \begin{array}{ll} \begin{aligned} & ^{CFD} D^{\varpi }_tu(t) = K, \;\;\;0<\varpi \le 1,\;\;t>0, \\ & u(0)= \,u_o. \end{aligned} \end{array}\right. } \end{aligned}$$

If we apply the Laplace transform (5) on both sides of the second model (23) we obtain

$$s^{\varpi }\mathcal {L}[u(t)](s)-s^{\varpi -1}u(0)=K\mathcal {L} [1, s],$$

and solving for $\mathcal {L}[u(t)](s)$ yields

$$\mathcal {L}[u(t)](s)=\frac{u_0}{s}+\frac{K\mathcal {L} [1, s]}{s^{\varpi }}.$$

Then applying the inverse Laplace transform we get

24 $$\begin{aligned} u(t)=u_0+\frac{K t^{\varpi }}{\Gamma {(\varpi +1)}}. \end{aligned}$$

The solution (24) is equivalent to

$$u(t)=u_o+\frac{{K}}{\Gamma (\varpi )}\int _0^t(t-{\tau })^{\varpi -1}d{\tau },$$

when we apply the anti-derivative on both sides of (23). Furthermore in order to obtain an average equal to 1, we consider

$$\frac{1}{\Gamma {(\varpi )}}=1,$$

which leads to

$$\Gamma {(\varpi )}=\Gamma {(1)}=\Gamma {(0)}=1.$$

**Fig. 4:**
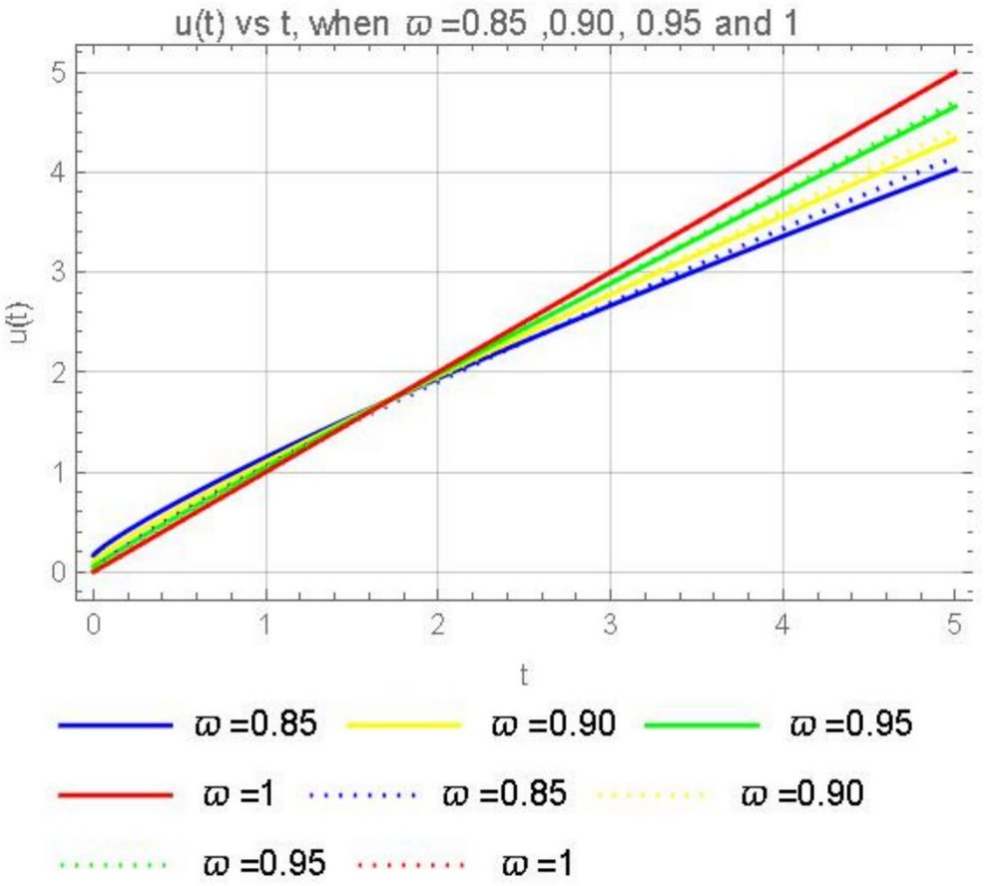
Numerical solutions of (14) and (23) with the initial condition $u_0=0$, when $K=1$, for $\varpi = 0.85$, 0.90, 0.95 and 1.

(i) . When $K=0,$ we recover from (24) the solution $u(t)=u_0=C$.
(ii) . When $\varpi =1,$ we get from (24) the well-known classical solution: $u(t)=u_{o} +Kt$. A graphical representation of the solutions for ABCFD and CFD second models is given in Fig. 4.

### Fractional relaxation model

Consider the third model in the form of the CFD

25 $$\begin{aligned} {\left\{ \begin{array}{ll} \begin{aligned} & ^{CFD} D^{\varpi }_tu(t) = -Ku, \;\;\;0<\varpi \le 1,\;\;t>0, \\ & u(0)= \,u_o. \end{aligned} \end{array}\right. } \end{aligned}$$

If we apply the Laplace transform (5) on both sides of the third model (25) we obtain

$$s^{\varpi }\mathcal {L}[u(t)](s)-s^{\varpi -1}u(0)=-K\mathcal {L}[u(t)](s),$$

and solving for $\mathcal {L}[u(t)](s)$ yields

$$\mathcal {L}[u(t)](s)= \frac{u_o s^{\varpi -1}}{s^{\varpi }+K}.$$

Applying the inverse Laplace transform we get

26 $$\begin{aligned} u(t)=u_o\mathcal {L}^{-1}\left[ \frac{s^{\varpi -1}}{s^{\varpi }+K}\right] . \end{aligned}$$

Since the Laplace transform of the Mittag-Leffler function is given by

27 $$\begin{aligned} \mathcal {L}\left[ t^{\alpha -1}E_{{\varpi }, {\alpha }}( \lambda t^{\varpi })\right] (s)=\frac{s^{\varpi -\alpha }}{s^\varpi -\lambda }, \end{aligned}$$

if $\alpha =1$ we have

28 $$\begin{aligned} \mathcal {L}[E_{{\varpi }, {1}}( \lambda t^{\varpi })](s)=\frac{s^{\varpi -1}}{s^\varpi -\lambda }, \end{aligned}$$

then it is clear that in (26)

$$\lambda =K,$$

so that solution in (26) when using (28) it becomes,

29 $$\begin{aligned} u(t)=u_o E_{{\varpi }, {1}}(-K t^{\varpi }). \end{aligned}$$

(i) . When $\varpi =1,$ we obtain from Eq. (29) the classical solution $u(t)=u_0 E_{1,1} (-Kt)=u_0e^{-Kt}.$
(ii) . When $K=0$, we have $u(t)=u_0$.

**Fig. 5:**
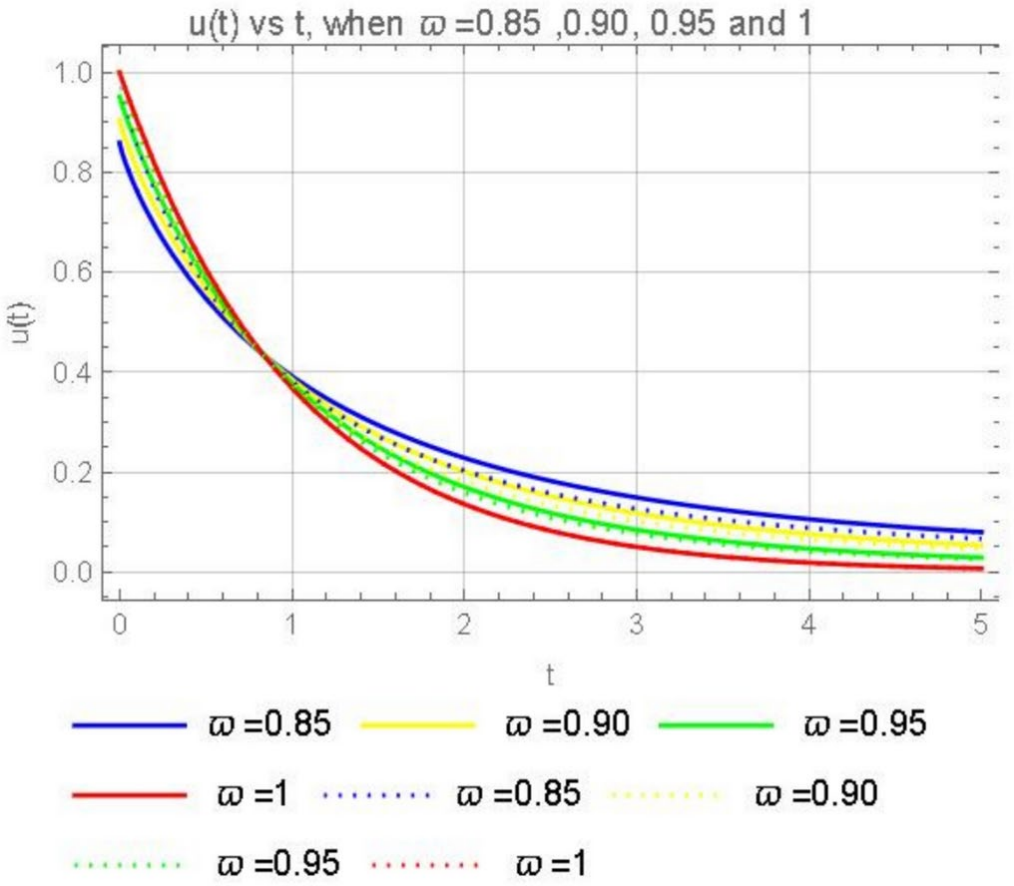
Numerical solutions (20) and (29) with the initial condition $u_0=1$ when $K=1$, $\varpi =0.85,$ 0.90, 0.95 and 1.

A graphical representation for the solutions (20) and (29) of the third models is given in Fig. 5.

Based on the Figs. 4 and 5 where the dotted lines represent the Caputo fractional derivative solutions and the solid lines represent the ABC (Atangana–Baleanu–Caputo) fractional derivative solutions, we can draw the following conclusions: The solid lines (ABC fractional derivative) and the dotted lines (Caputo fractional derivative) show how the solutions differ for the same values of $\varpi$. This comparison highlights the differences in the behavior of the two types of fractional derivatives. As $\varpi$ increases from 0.85 to 1, the solutions for both types of fractional derivatives converge towards the same behavior. This indicates that for $\varpi =1$, both fractional derivatives reduce to the classical integer-order derivative, resulting in the same solution. The solutions for different values of $\varpi$ show consistent patterns, this suggests that both fractional derivatives are stable and converge to the expected behavior as $\varpi$ approaches 1.

## Solving diffusion equation using separation of variables

### Example 1

Consider a one-dimensional fractional diffusion equation in the Atangana-Baleanu-Caputo sense for $t>0,\quad 0<\varpi \le 1,\quad x>0$ of the form:

30 $$\begin{aligned} {\left\{ \begin{array}{ll} \begin{aligned} ^{ABCFD} D_t^\varpi u(t, x)& =\Delta u(t, x), \\ u(0, x)& =f(x). \end{aligned} \end{array}\right. } \end{aligned}$$

By letting $g(t, x)= X(x)T(t)$ and utilizing the separation of variables method, then making substitutions in (30) yields

$$\frac{ ^{ABC F D} D_t^\varpi T(t)}{T(t)}=\frac{\Delta X(x)}{X(x)}.$$

Then let $-\lambda =\frac{ ^{ABCFD} D_t^\varpi T(t)}{T(t)}=\frac{\Delta X(x)}{X(x)}$, so that we obtain the following system

31 $$\begin{aligned} \Delta X(x)=-\lambda X(x), \end{aligned}$$

32 $$\begin{aligned} ^{ABCFD} D_t^\varpi T(t)=-\lambda T(t). \end{aligned}$$

We use an infinite sequence of pairs $\left\{ \delta _k, \psi _k\right\} _{k \in \mathbb {N}}$ to solve the eigenvalue system (31). $\left\{ \delta _k\right\}$ is an increasing sequence so that $\delta _k \rightarrow \infty$ and $\left\{ \psi _k\right\}$ is a family of functions that form a complete orthogonal set in $L^2\left( \left( x_0, \infty \right) \right)$^25,26^. Exploiting $\delta _k$ defined from (31), we can solve the eigenvalue issue (32) by letting $\lambda =\delta _k$. The following solution

33 $$\begin{aligned} T(t)=\frac{T_o B(\varpi )}{B(\varpi )+\lambda -\lambda \varpi }E_{\varpi , 1}\left( \frac{-\lambda \varpi t^\varpi }{B(\varpi )+\lambda -\lambda \varpi }\right) , \end{aligned}$$

is the unique solution of the eigenvalue problem

$$\begin{aligned}& ^{ABCFD} D_t^\varpi T(t)=-\lambda T(t), \quad t>0, \\&T(0)=T_0 . \end{aligned}$$

Then, the solution to Eq. (32) is provided as

$$T(t)=\tilde{f}(k) \frac{T_o B(\varpi )}{B(\varpi )+\lambda -\lambda \varpi }E_{\varpi , 1} \left( \frac{-\lambda \varpi t^\varpi }{B(\varpi )+\lambda -\lambda \varpi } \right) ,$$

where $\tilde{f}(k)$ is selected to fulfil the initial condition *f*(*x*). So that the solution of the initial value problem is given by

$$u(t, x)=\sum _{k=1}^{\infty } \tilde{f}(k) T(t) \psi _k(x).$$

### Example 2

Suppose that the fractional diffusion equation is in the form of the classical Caputo sense so that we have

34 $$\begin{aligned} {\left\{ \begin{array}{ll} \begin{aligned} ^{CFD} D_t^\varpi u(t, x)& =\Delta u(t, x), \\ u(0, x)& =f(x). \end{aligned} \end{array}\right. } \end{aligned}$$

Using the same method as in Example 1 we find the solution to be

$$u(t, x)=\sum _{k=1}^{\infty } \tilde{f}(k) T(t) \psi _k(x),$$

where $$T(t)=T_o E_{{\varpi }, {1}}({-\lambda } t^{\varpi }).$$

In both examples, it is clear that if we set the fractional parameter $\varpi$ equal to one, we obtain the solution of the classical diffusion equation of the integer-order derivative.

## HPSTM with the ABCFD

In this section, we discuss the homotopy perturbation Sumudu transform method (HPSTM). The basic concept of this technique is demonstrated as follows. Consider the general nonlinear fractional partial differential equation as^27–29,31^

35 $$\begin{aligned} ^{ABCFD}D^{\varpi }_t u(x, t)=R u(x, t)+N u(x, t)+g(x, t),\quad 0<\varpi \leqslant 1, \end{aligned}$$

$$u(x, 0)=f(x) \text{, }$$

where $^{ABCFD}D^{\varpi }_t u(x, t)$ is the Atangana-Baleanu fractional derivative in the Caputo sense of a function *u*(*x*, *t*),  *R* denotes the linear differential operator, *N* represents the general nonlinear differential operator and *g*(*x*, *t*) is the term arising from the source.

If the Sumudu transform of the ABCFD is applied on both sides of (35), we acquire:

36 $$\begin{aligned} \mathcal {S}\left[ ^{ABCFD}D^{\varpi }_t u(x, t)\right] =\mathcal {S}[R u(x, t)]+\mathcal {S}[N u(x, t)]+\mathcal {S}[g(x, t)]. \end{aligned}$$

Utilizing the property of the Sumudu transform of the ABCFD and the provided initial condition, we acquire

37 $$\begin{aligned} \mathcal {S}[u(x, t)]=f(x)+\left[ \frac{1-\varpi (1-s^{\varpi })}{B(\varpi )}\right] \mathcal {S} [g(x, t)]+\left[ \frac{1-\varpi (1-s^{\varpi })}{B(\varpi )}\right] \mathcal {S}[R u(x, t)+N u(x, t)]. \end{aligned}$$

If the inverse Sumudu transform is applied on both sides of (37), we acquire

38 $$\begin{aligned} u(x, t)=G(x, t)+\mathcal {S}^{-1}\left[ \left[ \frac{1-\varpi (1-s^{\varpi })}{B(\varpi )}\right] \mathcal {S} [R u(x, t)+N u(x, t)]\right] , \end{aligned}$$

where $G(x, t)=\mathcal {S}^{-1}\left( f(x)+\left[ \frac{1-\varpi (1-s^{\varpi })}{B(\varpi )}\right] \mathcal {S} [g(x, t)]\right)$ denotes the component arising from the source term and the given initial conditions. Then we create the following homotopy

39 $$\begin{aligned} u(x, t)=G(x, t)+q\left( \mathcal {S}^{-1}\left[ \mathcal {S}[R u(x, t)+N u(x, t)]\right] \right) . \end{aligned}$$

According to the homotopy perturbation method (HPM), we utilize the homotopy parameter *q* in order to expand the following solution

40 $$\begin{aligned} u(x, t)=\sum _{k=0}^{\infty } q^k u_k(x, t), \end{aligned}$$

and the nonlinear term *Nu*(*x*, *t*) is expanded utilizing the He’s polynomial as follows

41 $$\begin{aligned} N u(x, t)=\sum _{k=0}^{\infty } q^k H_k(u), \end{aligned}$$

where $H_k(u)$ are the He’s polynomials provided as^27–30^

42 $$\begin{aligned} \begin{aligned}&H_k\left( u_0, u_1, \ldots , u_k\right) =\frac{1}{k !} \frac{\partial ^k}{\partial q^k}\left[ N\left( \sum _{i=0}^{\infty } q^i u_i\right) \right] _{q=0}, \\&k=0,1,2,3, \ldots \end{aligned} \end{aligned}$$

By substituting Eqs. (40) and (41) in Eq. (39), yields

43 $$\begin{aligned} \begin{aligned}&\sum _{k=0}^{\infty } q^k u_k(x, t) \\&=G(x, t)+q\left( \mathcal {S}^{-1}\left[ \left[ \frac{1-\varpi (1-s^{\varpi })}{B(\varpi )}\right] \mathcal {S}\left[ R \sum _{k=0}^{\infty } q^k u_k(x, t)+\sum _{k=0}^{\infty } q^k H_k(u)\right] \right] \right) . \end{aligned} \end{aligned}$$

The following approximations are acquired after comparing the coefficients of the same powers of *q*.

$$\begin{aligned}&q^0: u_0(x, t)=G(x, t), \\&q^1: u_1(x, t)=\mathcal {S}^{-1}\left[ \left[ \frac{1-\varpi (1-s^{\varpi })}{B(\varpi )}\right] S\left[ R u_0(x, t)+H_0(u)\right] \right] , \\&q^2: u_2(x, t)=\mathcal {S}^{-1}\left[ \left[ \frac{1-\varpi (1-s^{\varpi })}{B(\varpi )}\right] S\left[ R u_1(x, t)+H_1(u)\right] ,\right. \\&q^3: u_3(x, t)=\mathcal {S}^{-1}\left[ \left[ \frac{1-\varpi (1-s^{\varpi })}{B(\varpi )}\right] S\left[ R u_2(x, t)+H_2(u)\right] \right] ,\\ \vdots \end{aligned}$$

Thus, we can write the solution of Eq. (35) as the following series:

$$u(x, t)=\operatorname {Lim}_{N \rightarrow \infty } \sum _{k=0}^N u_k(x, t) .$$

### Numerical examples

In this subsection, we provide the numerical examples for the two- and three-dimensional diffusion equations with the fractional order $\varpi$ specified as the Atangana-Baleanu-Caputo fractional order. The homotopy perturbation method coupled with the Sumudu transform is utilized to find the approximate and exact solutions.

#### Example 3

Consider the following 2-dimensional diffusion equation, with $\varpi$ specified as the Atangana-Baleanu-Caputo fractional order.

44 $$\begin{aligned} \begin{aligned}&\frac{\partial ^{\varpi } u}{\partial t^{\varpi }}=\frac{\partial ^2 u}{\partial x^2}+\frac{\partial ^2 u}{\partial y^2},\quad 0< \varpi \le 1,\quad 0\le x,y\le 1,\quad t>0, \end{aligned} \end{aligned}$$

and the following initial condition

$$u(x, y, 0)=x e^y .$$

Applying the Sumudu transform of the ABCFD on both sides of Eq. (44) and utilizing the above initial condition, we acquire

$$\mathcal {S}[u(x, y, t)]=x e^y+\left[ \frac{1-\varpi (1-s^{\varpi })}{B(\varpi )}\right] \mathcal {S}\left[ \frac{{\partial }^{2} u }{\partial x^2}+\frac{{\partial }^{2} u }{\partial y^2}\right] .$$

Using the inverse of the Sumudu transform we get

$$u(x, y, t)=x e^y+\mathcal {S}^{-1}\left[ \left[ \frac{1-\varpi (1-s^{\varpi })}{B(\varpi )}\right] \mathcal {S}\left[ \frac{{\partial }^{2} u }{\partial x^2}+\frac{{\partial }^{2} u }{\partial y^2}\right] \right] .$$

Applying the homotopy perturbation method (HPM), we obtain

$$\begin{aligned} \sum _{k=0}^{\infty } q^k u_k(x, y, t)&= x e^y +q\left( \mathcal {S} ^ { - 1 } \left[ \left[ \frac{1-\varpi (1-s^{\varpi })}{B(\varpi )}\right] \mathcal {S} \left[ \frac{{\partial }^{2} }{\partial x^2}\left( \sum _{k=0}^{\infty } q^k u_k(x, y, t)\right) \right. \right. \right. \\&\left. \left. \left. +\frac{{\partial }^{2} }{\partial y^2}\left( \sum _{k=0}^{\infty } q^k u_k(x, y, t)\right) \right] \right] \right) . \end{aligned}$$

After we have compared the coefficients of similar powers of *q*, we acquire

$$\begin{aligned}&q^0: u_0(x, y, t)=x e^y, \\&q^1: u_1(x, y, t)=\mathcal {S}^{-1}\left[ \left[ \frac{1-\varpi (1-s^{\varpi })}{B(\varpi )}\right] \mathcal {S}\left[ \frac{{\partial }^{2} u_0 }{\partial x^2}+\frac{{\partial }^{2} u_0 }{\partial y^2}\right] \right] =x e^y \left[ \frac{(1-\varpi )}{B(\varpi )}+\frac{t^{\varpi }}{B(\varpi ) \Gamma (\varpi )}\right] , \\&q^2: u_2(x, y, t)=\mathcal {S}^{-1}\left[ \left[ \frac{1-\varpi (1-s^{\varpi })}{B(\varpi )}\right] \mathcal {S}\left[ \frac{{\partial }^{2} u_1 }{\partial x^2}+\frac{{\partial }^{2} u_1 }{\partial y^2}\right] \right] =x e^y\left[ \frac{(1-\varpi )}{B(\varpi )}+\frac{t^{\varpi }}{B(\varpi ) \Gamma (\varpi )}\right] ^2, \\&q^3: u_3(x, y, t)=\mathcal {S}^{-1}\left[ \left[ \frac{1-\varpi (1-s^{\varpi })}{B(\varpi )}\right] \mathcal {S}\left[ \frac{{\partial }^{2} u_2 }{\partial x^2}+\frac{{\partial }^{2} u_2 }{\partial y^2}\right] \right] =x e^y \left[ \frac{(1-\varpi )}{B(\varpi )}+\frac{t^{\varpi }}{B(\varpi ) \Gamma (\varpi )}\right] ^3, \\&q^4: u_4(x, y, t)=\mathcal {S}^{-1}\left[ \left[ \frac{1-\varpi (1-s^{\varpi })}{B(\varpi )}\right] \mathcal {S}\left[ \frac{{\partial }^{2} u_3 }{\partial x^2}+\frac{{\partial }^{2} u_3 }{\partial y^2}\right] \right] =x e^y \left[ \frac{(1-\varpi )}{B(\varpi )}+\frac{t^{\varpi }}{B(\varpi ) \Gamma (\varpi )}\right] ^4, \end{aligned}$$

The homotopy perturbation Sumudu transform method series solution is as

45 $$\begin{aligned} \begin{aligned} u(x, y, t)&=x e^y\left( 1+\left[ \frac{(1-\varpi )}{B(\varpi )}+\frac{ t^{\varpi }}{B(\varpi ) \Gamma (\varpi )}\right] +\left[ \frac{(1-\varpi )}{B(\varpi )}+\frac{ t^{\varpi }}{B(\varpi ) \Gamma (\varpi )}\right] ^2+\left[ \frac{(1-\varpi )}{B(\varpi )}+\frac{ t^{\varpi }}{B(\varpi ) \Gamma (\varpi )}\right] ^3+\dots \right) . \end{aligned} \end{aligned}$$

So, the expanded form is:

46 $$\begin{aligned} \begin{aligned}&u(x, y, t)=x e^y \left( 1 +\left[ \frac{(1-\varpi )}{B(\varpi )}+\frac{ t^{\varpi }}{B(\varpi ) \Gamma (\varpi )}\right] +\left[ \frac{(1-\varpi )^2}{(B(\varpi ))^2}+\frac{2(1-\varpi ){ t^{\varpi }}}{(B(\varpi ))^2 \Gamma (\varpi )}+\frac{ t^{2\varpi }}{(B(\varpi ))^2 \Gamma (2\varpi )}\right] \right. \\&+\left[ \frac{(1-\varpi )^3}{(B(\varpi ))^3}+\frac{3(1-\varpi )^2 t^{\varpi }}{(B(\varpi ))^3 \Gamma (\varpi )}+\frac{3(1-\varpi ) t^{2\varpi }}{(B(\varpi ))^3 \Gamma (2\varpi )}+\frac{ t^{3\varpi }}{(B(\varpi ))^3 \Gamma (3\varpi )}\right] +\dots \dots ). \end{aligned} \end{aligned}$$

If we using the Gamma property $\Gamma (x)=\frac{\Gamma (x+1)}{x},$ Eq. (46) can also be written as

$$\begin{aligned} u(x, y, t)=&x e^y \left( 1 +\left[ \frac{(1-\varpi )}{B(\varpi )}+\frac{{\varpi } t^{\varpi }}{B(\varpi ) \Gamma (\varpi +1)}\right] +\left[ \frac{(1-\varpi )^2}{(B(\varpi ))^2}+\frac{2(1-\varpi ){{\varpi } t^{\varpi }}}{(B(\varpi ))^2 \Gamma (\varpi +1)}+\frac{ {2\varpi }t^{2\varpi }}{(B(\varpi ))^2 \Gamma (2\varpi +1)}\right] \right. \\&+\left[ \frac{(1-\varpi )^3}{(B(\varpi ))^3}+\frac{3(1-\varpi )^2 {\varpi }t^{\varpi }}{(B(\varpi ))^3 \Gamma (\varpi +1)}+\frac{3(1-\varpi ) {2\varpi }t^{2\varpi }}{(B(\varpi ))^3 \Gamma (2\varpi +1)}+\frac{{3\varpi }t^{3\varpi }}{(B(\varpi ))^3 \Gamma (3\varpi +1)}\right] +\dots \dots ). \end{aligned}$$

Setting $\varpi =1$ in Eq. (46), we get

47 $$\begin{aligned} u(x, y, t)=x e^y\left( 1+t+\frac{t^2}{2 !}+\frac{t^3}{3 !}+\frac{t^4}{4 !}+\cdots \right) = x e^{y}\sum ^\infty _{k=0}\frac{t^k}{k !}. \end{aligned}$$

Equation (47) is equivalent to the closed-form exact solution of the form

48 $$\begin{aligned} u(x, y, t)= x e^{y+t}. \end{aligned}$$

**Fig. 6:**
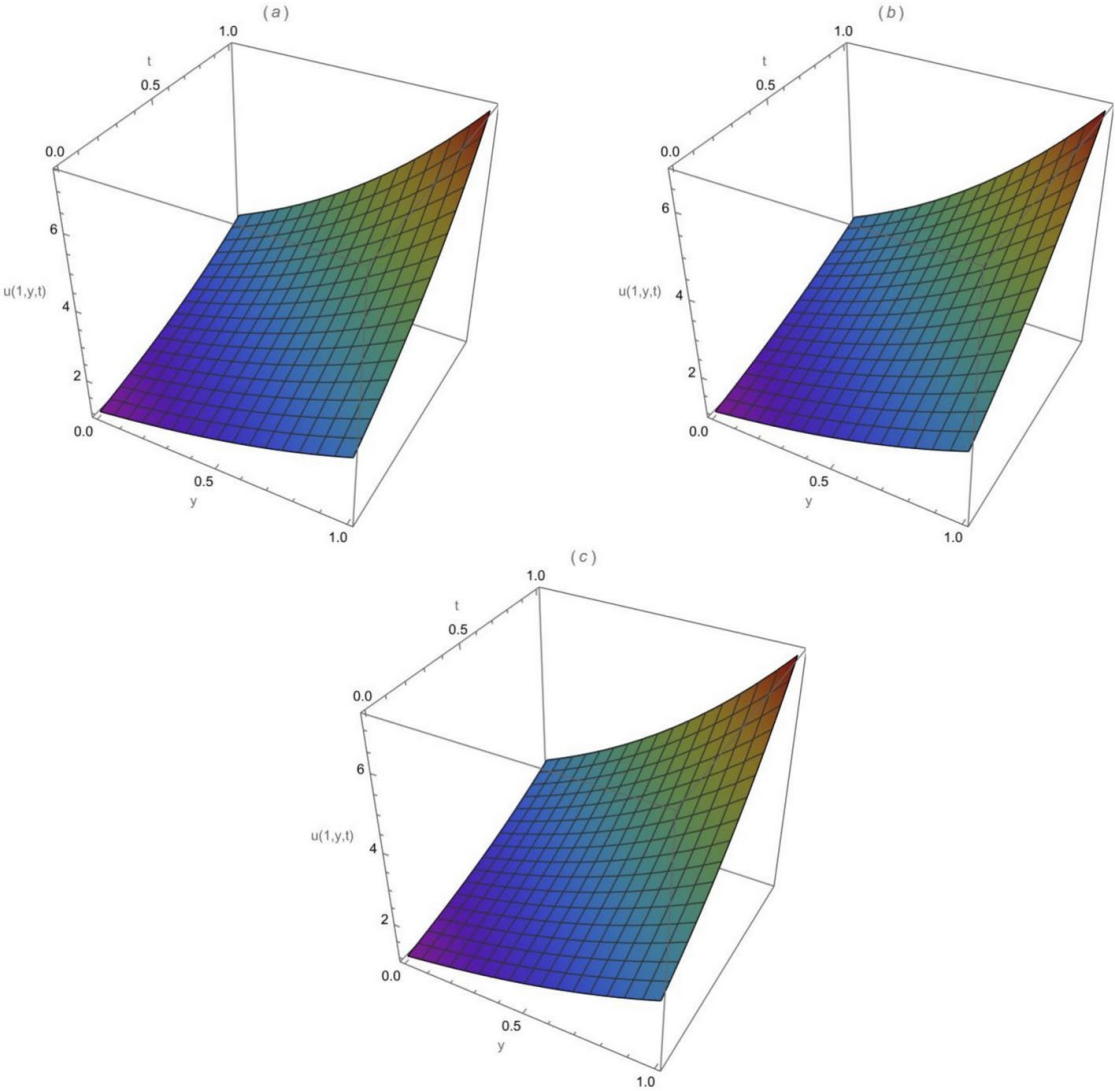
(**a**) Exact solution (48) when $x=1$, (**b**) approximate solution (47) when $\varpi =1$, $x=1$ and k=2, and (**c**) the approximate solution (47) when $\varpi =1$, $x=1$ and k=3.

The behaviours of the exact solution (48) and the approximate solution (47) are illustrated in Fig. 6(a)-(c): (a) is the surface for the exact solution (48) when $x=1$, (b) is the surface for the approximate solution (47) when $\varpi =1,$ $x=1$ and k=2 and (c) is the surface for the approximate solution (47) when $\varpi =1,$ $x=1$ and k=3. It is easy to observe from Fig. 6 that the approximate solutions found using the HPSTM are close to the exact solution when we increase the computing term *k*. In addition, the effectiveness of the current technique can be dramatically improved by determining more components of *u*(*x*, *y*, *t*).

**Fig. 7:**
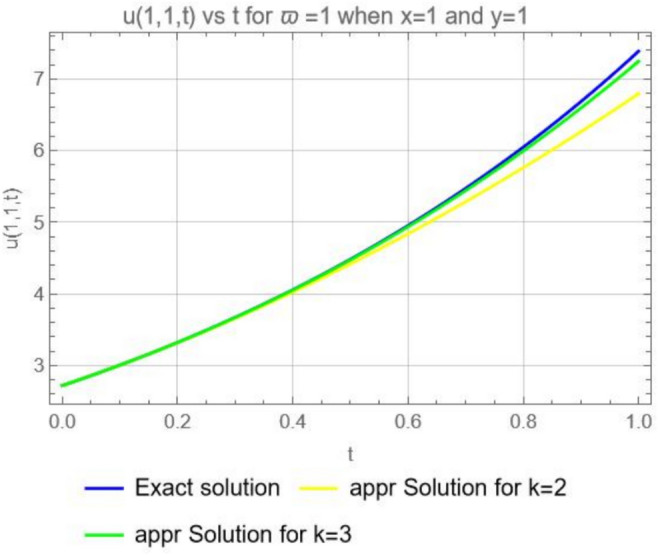
Exact solution (48) and approximate solution (47) when $x=1$, $y=1$ for $\varpi =1$, $k=2$ and $k=3$.

Figure 7, illustrates the graphical representations for the exact solution (48) and approximate solutions (47), when $x=1$, $y=1$, and $\varpi =1$, $k=2$, $k=3$. We can observe that, if we increase the computing term *k* from 2 to 3 the approximate solutions get closer to the exact solution, hence the relative errors will be very small for the approximate solutions with the increase in *k*.

**Fig. 8:**
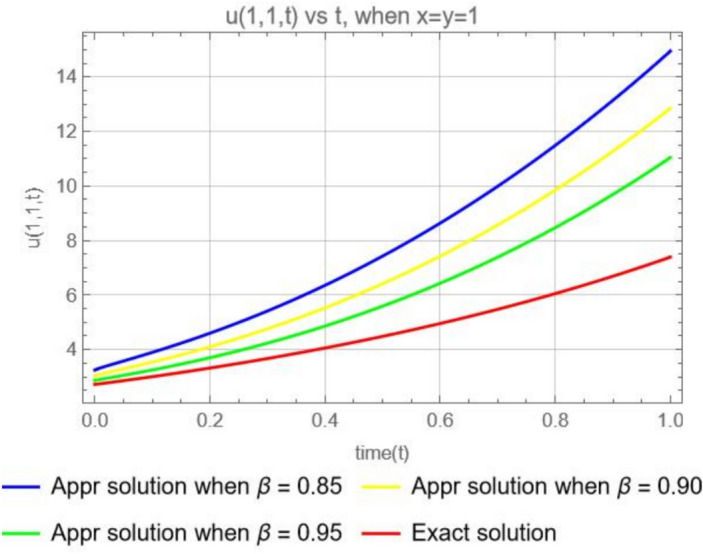
The plot shows the function $u_3(1,1,t)$ for different values of $\beta$ (0.85, 0.90, 0.95) and exact solution.

Figure 8, illustrates the graphical representations for the fractional 3rd terms approximate solution (46), when $x=1$, $y=1$, with different parameters $\varpi =0.85,0.90, 0.95$ and the exact solution (48). We can observe that, As the fractional order $(\beta )$ increases, the approximate solutions tend to get closer to the exact solution (48). This is because higher values of fractional parameter $(\beta )$ generally improve the accuracy of the approximation.

Suppose that the 2-dimensional diffusion equation (44) is in the form of the CFD, then we have the solution

49 $$\begin{aligned} u(x, y, t)=xe^{y} \sum ^\infty _{k=0}\frac{ t^{k\varpi }}{ \Gamma (k\varpi +1)}=x e^y\left( 1+\frac{t^{\varpi }}{\Gamma (\varpi +1)}+\frac{t^{2\varpi }}{ \Gamma (2\varpi +1)}+\frac{ t^{3\varpi }}{ \Gamma (3\varpi +1)}+\cdots \right) . \end{aligned}$$

Or

50 $$\begin{aligned} u(x, y, t)=x e^y\left( 1+\frac{t^{\varpi }}{\varpi \Gamma (\varpi )}+\frac{t^{2\varpi }}{2\varpi \Gamma (2\varpi )}+\frac{ t^{3\varpi }}{3\varpi \Gamma (3\varpi )}+\cdots \right) . \end{aligned}$$

**Fig. 9:**
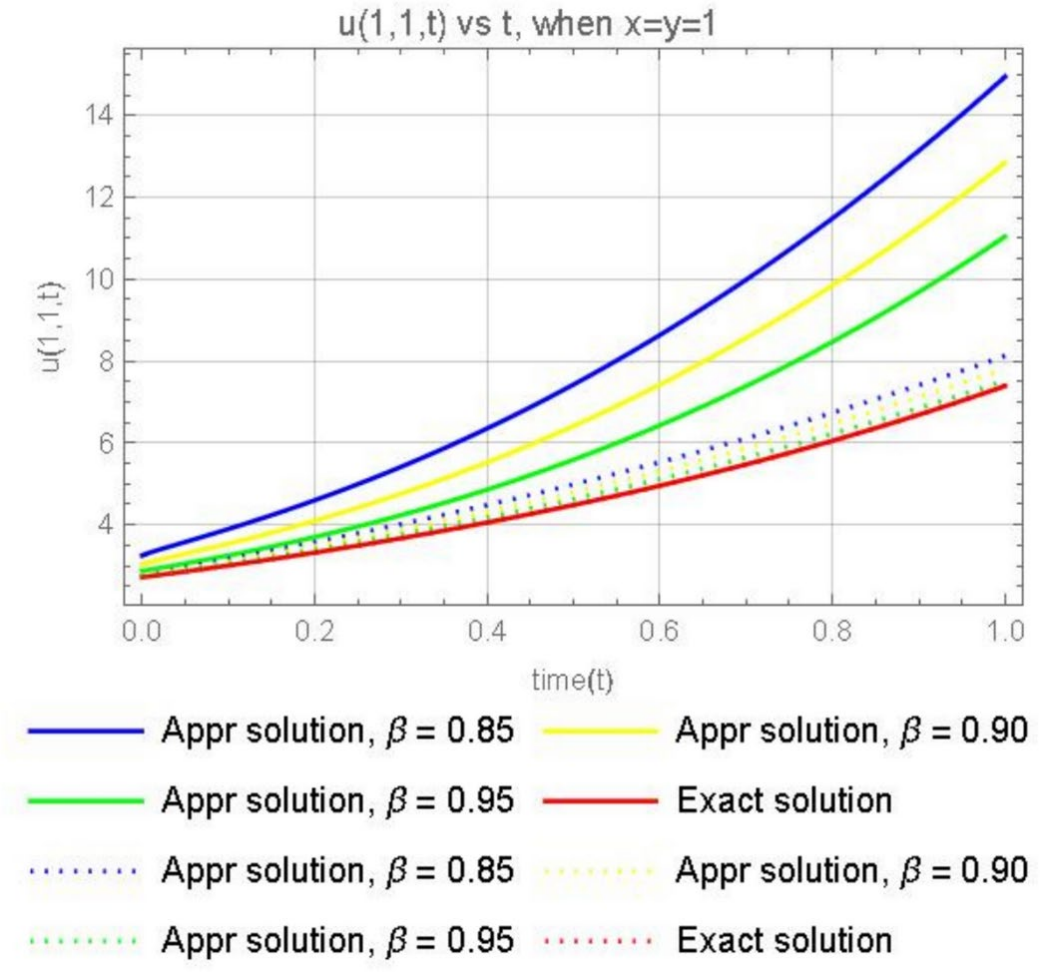
Comparison of the ABCFD solution with the CFD solution for different values of $\beta$ (0.85, 0.90, 0.95).

In Fig. 9, where the dotted line represents the CFD solutions and the solid line represents the ABCFD solution, we observe that the faster convergence of the Caputo fractional derivative solutions suggests they may be more suitable for this particular system, providing accurate and efficient approximations. However, the choice between the Caputo and ABC fractional derivatives should still consider the specific physical characteristics of the system and the importance of non-local and memory effects.

Below we provide the formulas for absolute error $_{absolute}E_{k}(u)$ and relative error $_{relative}E_{k}(u)$

51 $$\begin{aligned} _{absolute}E_{k}(u)=\left| u_{exact}-u_{\text{ approximate } }\right| \end{aligned}$$

52 $$\begin{aligned} _{relative}E_{k}(u)=\frac{\left| u_{exact}-u_{\text{ approximate } }\right| }{|u_{exact}|}\times {100} \end{aligned}$$

Table 1 below we compare the exact solution (48) and 10th term approximate solution (47), when $\varpi =1$, $x=\frac{1}{2}$ and $t=\frac{1}{2}$, with different values of *y*.

**Table 1:** Comparison of the solutions using absolute and relative errors.

*y*	Exact solution	Approximate solution	$_{absolute}E_{10}(u)$	$_{relative}E_{10}(u)$
0	0.824360635	0.824360635	0	0
0.2	1.006876353	1.006876353	0	0
0.4	1.229801556	1.229801556	0	0
0.6	1.502083012	1.502083012	0	0
0.8	1.834648333	1.834648333	0	0
1.0	2.240844535	2.240844535	0	0

In Table 1, we see that the approximate solutions at dissimilar grid points acquired by the homotopy perturbation Sumudu transform method are identical to the exact solution when computing the approximate solution up to the $10{\text{ th } }$ term.

#### Example 4

Consider the following 3-dimensional diffusion equation, with $\varpi$ specified as the Atangana-Baleanu-Caputo fractional order.

$$\begin{aligned}&\frac{\partial ^\varpi u}{\partial t^\varpi }=\frac{\partial ^2 u}{\partial x^2}+\frac{\partial ^2 u}{\partial y^2}+\frac{\partial ^2 u}{\partial z^2},\quad 0< \varpi \le 1,\quad 0\le x,y,z\le 1,\quad t>0, \\&\quad \text{ considering } \text{ the } \text{ following } \text{ initial } \text{ condition } \\&u(x, y, z, 0)=e^{x+y+z} \text{. } \\&\text{ Applying } \text{ the } \text{ homotopy } \text{ perturbation } \text{ Sumudu } \text{ transform } \text{ method } \text{(HPSTM), } \text{ we } \text{ obtain } \\&\sum _{\textrm{k}=0}^{\infty } q^k u_k(x, y, z, t)=e^{x+y+z}+q\left( \mathcal {S} ^ { - 1 } \left[ \left[ \frac{1-\varpi (1-s^{\varpi })}{B(\varpi )}\right] \mathcal {S}\left[ \frac{{\partial }^{2} }{\partial x^2}\left( \sum _{k=0}^{\infty } q^k u_k(x, y, z, t)\right) \right. \right. \right. \\&\left. \left. +\frac{{\partial }^{2} }{\partial y^2}\left( \sum _{k=0}^{\infty } q^k u_k(x, y, z, t)\right) +\frac{{\partial }^{2} }{\partial z^2}\left( \sum _{k=0}^{\infty } q^k u_k(x, y, z, t)\right) \right] \right) . \end{aligned}$$

After we have compared the coefficients of similar powers of *q*, we acquire

$$\begin{aligned}&q^0: u_0(x, y, z, t)=e^{x+y+z}, \\&q^1: u_1(x, y, z, t)= 3e^{x+y+z} \left[ \frac{(1-\varpi )}{B(\varpi )}+\frac{{\varpi } t^{\varpi }}{B(\varpi ) \Gamma (\varpi +1)}\right] , \\&q^2: u_2(x, y, z, t)=3^2e^{x+y+z} \left[ \frac{(1-\varpi )}{B(\varpi )}+\frac{{\varpi } t^{\varpi }}{B(\varpi ) \Gamma (\varpi +1)}\right] ^2, \\&q^3: u_3(x, y, z, t)= 3^3e^{x+y+z} \left[ \frac{(1-\varpi )}{B(\varpi )}+\frac{{\varpi } t^{\varpi }}{B(\varpi ) \Gamma (\varpi +1)}\right] ^3, \\&q^4: u_4(x, y, z, t)=3^4 e^{x+y+z}\left[ \frac{(1-\varpi )}{B(\varpi )}+\frac{{\varpi } t^{\varpi }}{B(\varpi ) \Gamma (\varpi +1)}\right] ^4, \end{aligned}$$

Then, the series solution obtained by the homotopy perturbation Sumudu transform method is given as

$$\begin{aligned} u(x, y, z, t)= e^{x+y+z}(1&+3\left[ \frac{(1-\varpi )}{B(\varpi )}+\frac{{\varpi } t^{\varpi }}{B(\varpi ) \Gamma (\varpi +1)}\right] \\&\left. +3^2\left[ \frac{(1-\varpi )}{B(\varpi )}+\frac{{\varpi } t^{\varpi }}{B(\varpi ) \Gamma (\varpi +1)}\right] ^2+\cdots \right) . \end{aligned}$$

So that when using the series expansion $\frac{1}{1-x}=1+x+x^2+x^3+\cdots =\sum ^\infty _{k=0}x^k$ we get the following fractional series solution

53 $$\begin{aligned} u(x, y, t)= e^{(x+y+z)} \sum ^\infty _{k=0}(3)^k\left[ \frac{(1-\varpi )}{B(\varpi )}+\frac{{\varpi } t^{\varpi }}{B(\varpi ) \Gamma (\varpi +1)}\right] ^k. \end{aligned}$$

Since

$$\left[ \frac{(1-\varpi )}{B(\varpi )}+\frac{t^{\varpi }}{B(\varpi ) \Gamma (\varpi )}\right] =\left[ \frac{(1-\varpi )}{B(\varpi )}+\frac{{\varpi } t^{\varpi }}{B(\varpi ) \Gamma (\varpi +1)}\right] ,$$

after using the Gamma property $\Gamma (x)=\frac{\Gamma (x+1)}{x},$ then the fractional series solution (53) can also be written as

$$u(x, y, t)= e^{(x+y+z)} \sum ^\infty _{k=0}(3)^k\left[ \frac{(1-\varpi )}{B(\varpi )}+\frac{ t^{\varpi }}{B(\varpi ) \Gamma (\varpi )}\right] ^k.$$

And we can use the binomial theorem, which states that:

$$(a+b)^k=\sum ^k_{n=0}\left( \begin{array}{c}k \\ n\end{array} \right) a^{k-n}b^n,$$

to expand the expression $\left[ \frac{(1-\varpi )}{B(\varpi )}+\frac{ t^{\varpi }}{B(\varpi ) \Gamma (\varpi )}\right] ^k$ or $\left[ \frac{(1-\varpi )}{B(\varpi )}+\frac{{\varpi } t^{\varpi }}{B(\varpi ) \Gamma (\varpi +1)}\right] ^k,$ taking in to account the Gamma property $\frac{(t^\varpi )^k}{(\Gamma (\varpi ))^k} =\frac{t^{k\varpi }}{\Gamma (k\varpi )}$ like in Example 3.

Setting $\varpi =1$ in Eq. (53), we obtain the classical series solution as

54 $$\begin{aligned} u(x, y, z, t)= e^{x+y+z}\left( 1+3t+\frac{(3t)^2}{2 !}+\frac{(3t)^3}{3 !}+\frac{(3 t)^4}{4 !}+\cdots \right) = e^{x+y+z}\sum ^\infty _{k=0}\frac{{(3t)}^k}{k !}. \end{aligned}$$

Equation (54) is equivalent to the closed form exact solution of the form

55 $$\begin{aligned} u(x, y, z, t)=e^{x+y+z+3t}. \end{aligned}$$

**Fig. 10:**
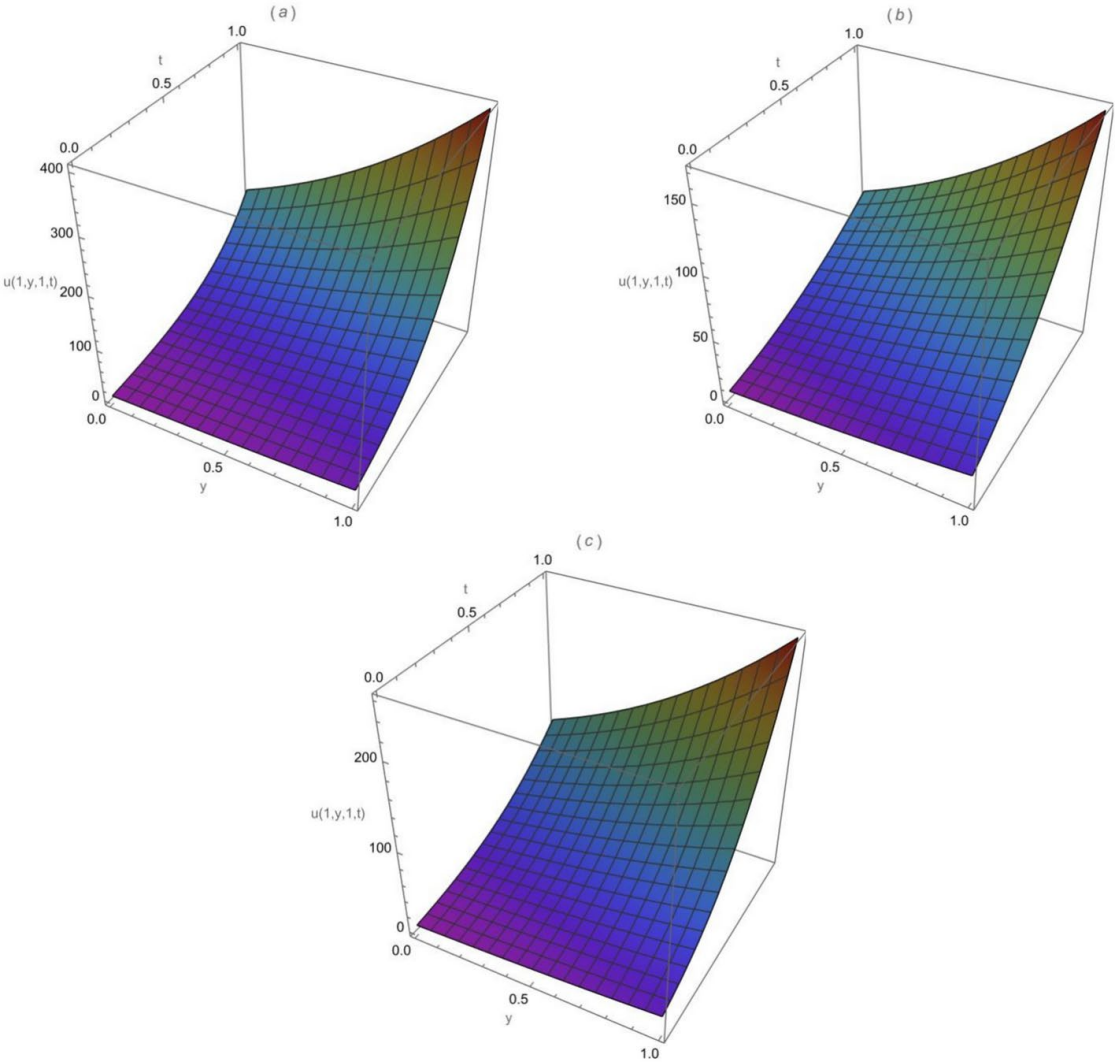
(**a**) Exact solution *u*(1, *y*, 1, *t*) , (**b**) approximate solution $u_2 (1, y, 1, t)$ and (**c**) approximate solution $u_3 (1, y, 1, t)$ when $\varpi =1$ , $x=1$ and $z=1$.

Figure 10 shows the surfaces of the solution *u*(*x*, *y*, *z*, *t*) when $\varpi =1$, $z=1$ and $x=1$: (a) is for the exact solution (55), (b) is for the approximate solution (54) when $k=2$, and (c) is for the approximate solution (54) when $k=3$. It is observed from Fig. 10 that *u*(1, *y*, 1, *t*) increases when there is an increase in *t* and *y*. Furthermore, the approximate solutions acquired by the homotopy perturbation Sumudu transform method are nearly the same as the exact solution especially when we increase the number of computing terms. Same as in Example 3 the second and third-order terms were used to get the approximation solutions. From Fig. 10, similarly we observe that the more we increase the computing terms, the closer we get to the exact solution, which means the efficiency of using this method can be dramatically improved by obtaining more terms of *u*(*x*, *y*, *z*, *t*).

**Fig. 11:**
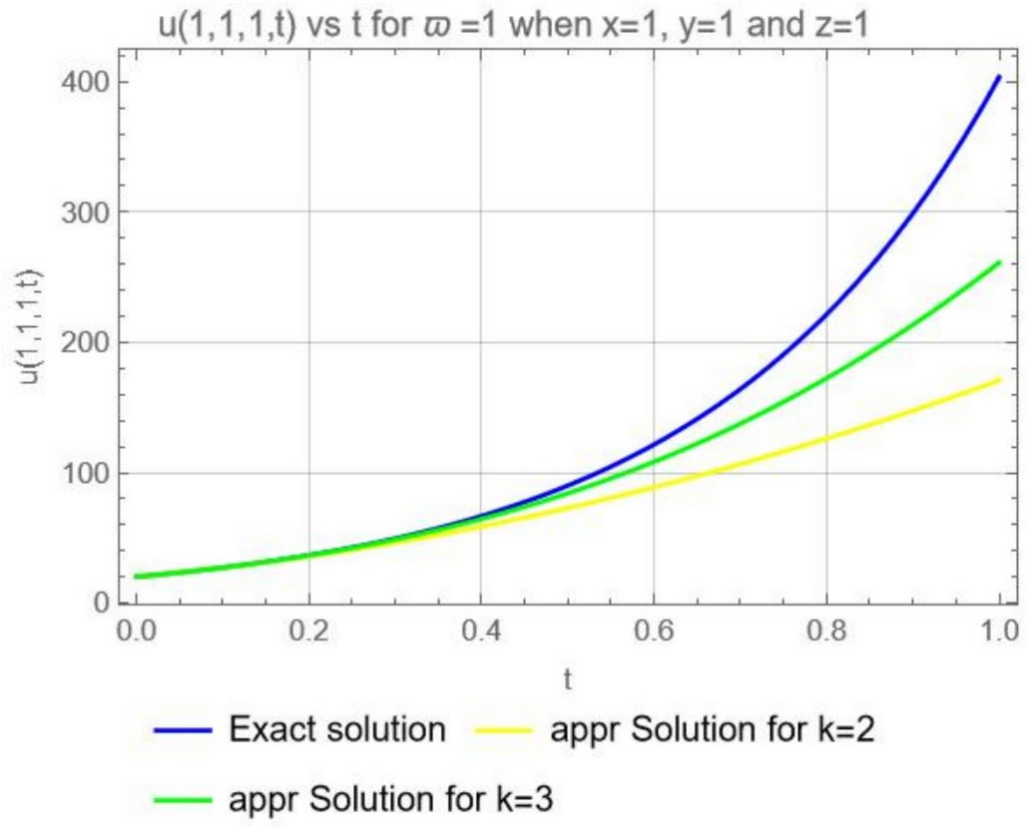
Exact solution *u*(1, 1, 1, *t*) and approximate solutions $u_2 (1, 1, 1, t)$, and $u_3 (1, 1, 1, t)$ when $\varpi =1$ , $x=1$ , $y=1$ and $z=1$.

Figure 11, illustrates the graphical representation for the exact solution (55) and approximate solution (54) when $\varpi =1$, $x=1$, $y=1$, $z=1$, for $k=2$ and $k=3$ and it is clear that if we increase the number of computing term the series converge to the exact solution (55).

**Fig. 12:**
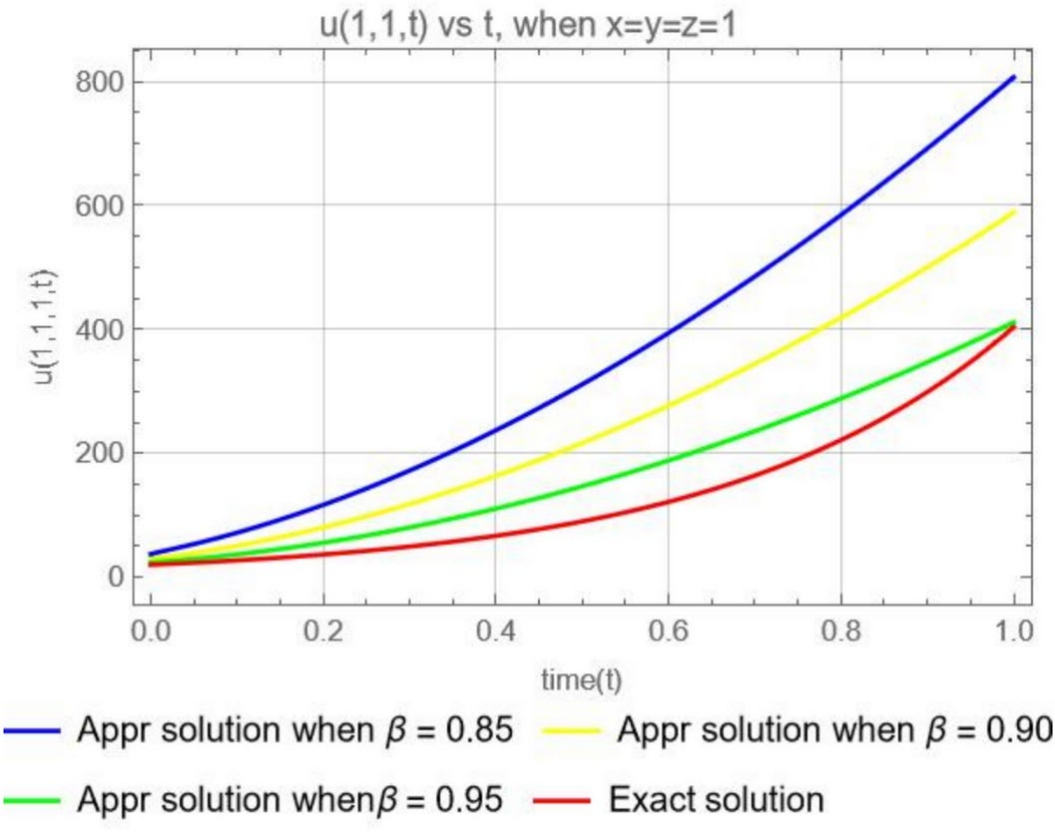
The plot shows the function $u_3(1,1,1,t)$ for different values of $\beta$ (0.85, 0.90, 0.95) and Exact solution.

Figure 12, illustrates the graphical representations for the 3rd terms approximate solution (53) , when $x=y=z=1$, with different parameters $\varpi =0.85,0.90, 0.95$ and the exact solution (55). We can reason the same way as in Example 3.

Suppose that the 3-dimensional diffusion equation in Example 4 is in the form of the CFD, then we have the solution

56 $$\begin{aligned} u(x, y, z, t)= e^{(x+y+z)} \sum ^\infty _{k=0}(3)^k\frac{ t^{k\varpi }}{ \Gamma (k\varpi +1)}= e^{x+y+z}\left( 1+\frac{3t^{\varpi }}{\Gamma (\varpi +1)}+\frac{3^2 t^{2\varpi }}{ \Gamma (2\varpi +1)}+\frac{3^3 t^{3\varpi }}{ \Gamma (3\varpi +1)}+\cdots \right) . \end{aligned}$$

Or

57 $$\begin{aligned} u(x, y, z, t)= e^{x+y+z}\left( 1+\frac{3t^{\varpi }}{\varpi \Gamma (\varpi )}+\frac{3^2 t^{2\varpi }}{ 2\varpi \Gamma (2\varpi )}+\frac{3^3 t^{3\varpi }}{3\varpi \Gamma (3\varpi )}+\cdots \right) . \end{aligned}$$

**Fig. 13:**
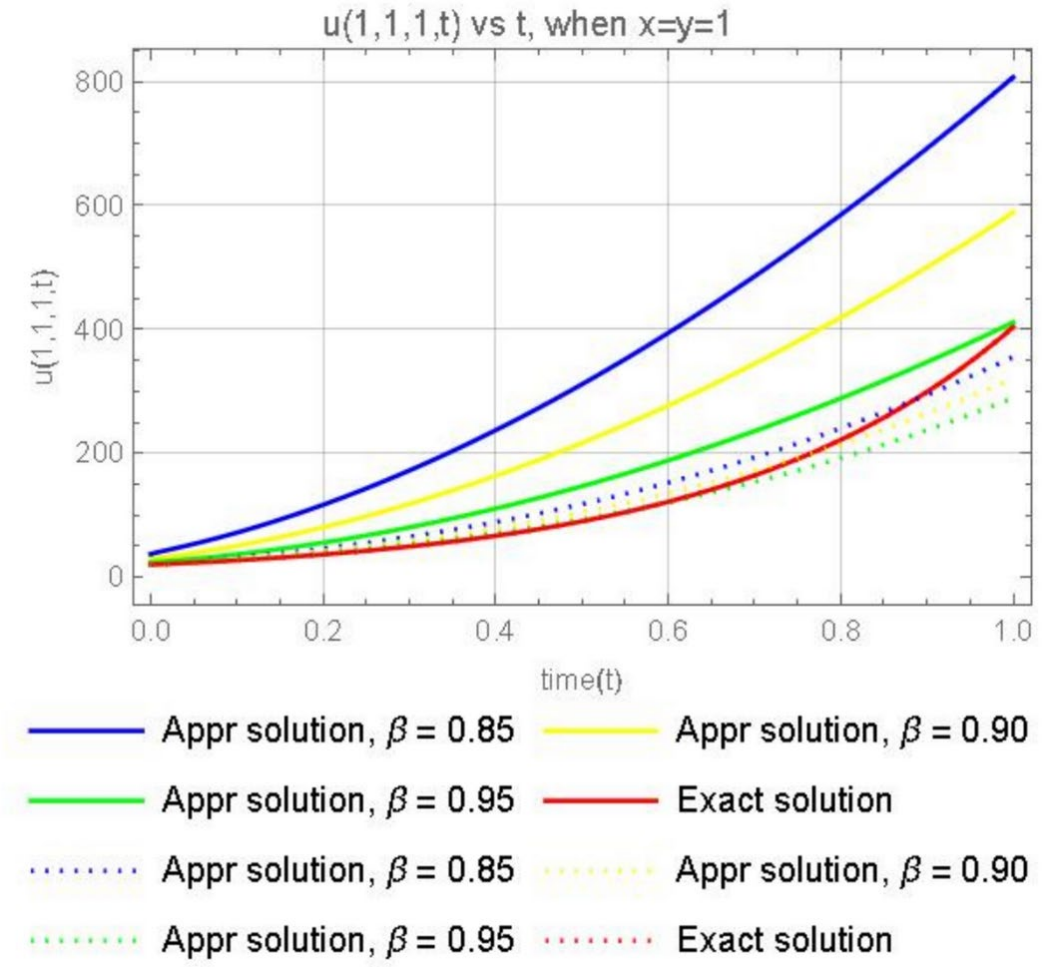
Comparison of the ABCFD solution with the CFD solution for different values of $\beta$ (0.85, 0.90, 0.95).

For Fig. 13 we can reason the same way as for Figure 9.

Table 2 comparison of the exact solution (55) and approximate solution (54), when $\varpi =1$, $x=\frac{1}{2}$, $z=\frac{1}{2}$ and $t=\frac{1}{2}$, with different values of *y*.

**Table 2:** Comparison of the solutions using absolute and relative errors.

*y*	Exact solution	Approximate solution	$_{absolute}E_{10}(u)$	$_{relative}E_{10}(u)$
0	12.18249396	12.18248724	$6.72 \times 10^{-6}$	$5.52 \times 10^{-5}$
0.2	14.87973172	14.87972351	$8.21 \times 10^{-6}$	$5.52 \times 10^{-5}$
0.4	18.17414537	18.17413534	$1.00 \times 10^{-5}$	$5.52 \times 10^{-5}$
0.6	22.19795128	22.19793903	$1.23 \times 10^{-5}$	$5.52 \times 10^{-5}$
0.8	27.11263892	27.11262396	$1.50 \times 10^{-5}$	$5.52 \times 10^{-5}$
1.0	33.11545196	33.11543369	$1.83 \times 10^{-5}$	$5.52 \times 10^{-5}$

In Table 2, we see that the approximate solutions at dissimilar grid points acquired by the homotopy perturbation Sumudu transform method are close to the exact solution when computing the approximate solution up to the $10^{\text{ th } }$ term.

Table 3 shows the comparison of fractional derivatives with various aspects such as the kernel used, convergence rate, and stability. Some information from the table can be found in^3,17^.

**Table 3:** Comparison of ABC, Caputo–Fabrizio, and classical Caputo fractional derivatives.

Parameter	ABC fractional derivative	Caputo–Fabrizio fractional derivative	Classical Caputo fractional derivative
Kernel used	Mittag-Leffler	Exponential	Power Law
Convergence rate	Moderate	Moderate	Fast
Accuracy	High for non-local effects	High for certain physical models	High in initial stages
Stability	High	High	High
Sensitivity to parameters	Low	Moderate	Moderate
Computational efficiency	Moderate	High	Moderate
Application context	Memory and hereditary systems	Viscoelastic materials, electrical circuits	Anomalous diffusion, control systems
Key findings	Captures non-local effects effectively	Suitable for modeling material heterogeneities	Fast convergence to exact solutions

## Conclusions and future work

In this paper, we have solved the three fractional kinetic models and showed that they all lead us to a classical derivative when the fractional order $\varpi = 1$. We used the method of separation of variables to find the solution to a one-dimensional fractional diffusion equation. We have successfully utilized the homotopy perturbation method coupled with the Sumudu transform of the Atangana-Baleanu-Caputo fractional derivative to find the exact and approximate solutions of the two and three-dimensional fractional diffusion equations. The technique delivered the solutions in the form of a power series, that contains easily computable terms which are converging to the exact solution. It was observed that when we increase the number of computing terms, the series converges to the exact solution so that the absolute error between the exact solution and the approximate solution becomes very small. The solutions found by the ABCFD method were compared with the solutions found by the CFD method. Our analysis reveals that the Classical Caputo method approaches the exact solution faster than the ABC method for the given problem. This suggests that the Caputo method may provide higher accuracy in the initial stages of $t_0$. Despite this, the ABC method’s unique properties, such as its ability to capture non-local effects, make it valuable for other types of problems. This provides enough evidence that the HPSTM is effective in terms of acquiring the solutions for different types of partial differential equations and many other equations that involve differential equations. The findings of this study can lead to new directions such as creating more precise and effective numerical techniques, like in^32^, the Variational Iteration Method is combined with the Elzaki transform, to solve fractional diffusion equations. We can combine the homotopy perturbation or Adomian decomposition method with the Elzaki transform (or other transforms) to create a new method to solve linear and nonlinear fractional differential equations. Applications of fractional diffusion equations are becoming more common in disciplines like biology, engineering, and physics. These equations are particularly useful in modeling processes that exhibit anomalous diffusion or memory effects, which are not adequately described by classical diffusion equations. Future studies will try to expand on these applications and develop new ones, potentially exploring areas such as financial mathematics, control theory, and materials science. Additionally, researchers will aim to improve the computational efficiency and accuracy of these methods, making them more accessible for practical use.

## Data Availability

All data generated or analysed during this study are included in this published article.
